# Relationships between aboveground biomass and plant cover at two spatial scales and their determinants in northern Tibetan grasslands

**DOI:** 10.1002/ece3.3308

**Published:** 2017-08-30

**Authors:** Yanbin Jiang, Yangjian Zhang, Yupeng Wu, Ronggui Hu, Juntao Zhu, Jian Tao, Tao Zhang

**Affiliations:** ^1^ Key Laboratory of Arable Land Conservation (Middle and Lower Reaches of Yangtze River) Ministry of Agriculture College of Resources and Environment Huazhong Agricultural University Wuhan China; ^2^ Lhasa Station Key Laboratory of Ecosystem Network Observation and Modeling Institute of Geographic Sciences and Natural Resources Research Chinese Academy of Sciences Beijing China; ^3^ Center for Excellence in Tibetan Plateau Earth Sciences Chinese Academy of Sciences Beijing China; ^4^ Tobacco Research Institute of Chinese Academy of Agricultural Sciences Qingdao China; ^5^ College of Agronomy Shenyang Agricultural University Shenyang China

**Keywords:** aboveground biomass, grassland, plant cover, precipitation, soil nitrogen content, species composition, species richness, Tibet

## Abstract

The relationships between cover and AGB for the dominant and widely distributed alpine grasslands on the northern Tibetan Plateau is still not fully examined. The objectives of this study are to answer the following question: (1) How does aboveground biomass (AGB) of alpine grassland relate to plant cover at different spatial scales? (2) What are the major biotic and abiotic factors influencing on AGB–cover relationship? A community survey (species, cover, height, and abundance) was conducted within 1 m × 1 m plots in 70 sites along a precipitation gradient of 50–600 m. Ordinary linear regression was employed to examine AGB–cover relationships of both community and species levels at regional scale of entire grassland and landscape scale of alpine meadow, alpine steppe, and desert steppe. Hierarchical partitioning was employed to estimate independent contributions of biotic and abiotic factors to AGB and cover at both scales. Partial correlation analyses were used to discriminate the effects of biotic and abiotic factors on AGB–cover relationships at two spatial scales. AGB and community cover both exponentially increased along the precipitation gradient. At community level, AGB was positively and linearly correlated with cover for all grasslands except for alpine meadow. AGB was also linearly correlated with cover of species level at both regional and landscape scales. Contributions of biotic and abiotic factors to the relationship between AGB and cover significantly depended on spatial scales. Cover of cushions, forbs, legumes and sedges, species richness, MAP, and soil bulk density were important factors that influenced the AGB–cover relationship at either regional or landscape scale. This study indicated generally positive and linear relationships between AGB and cover are at both regional and landscape scales. Spatial scale may affect ranges of cover and modify the contribution of cover to AGB. AGB–cover relationships were influenced mainly by species composition of different functional groups. Therefore, in deriving AGB patterns at different spatial scales, community composition should be considered to obtain acceptable accuracy.

## 1 INTRODUCTION

Aboveground biomass (AGB) is an important ecological property and is closely linked to nutrient cycle, energy flow, and carbon cycles. In grassland ecosystems, spatial variations in AGB were used to address forage availability, carbon balance, and responses to global climate change (Luo, Li, & Zhu, 2002; Yahdjian & Sala, 2006). Quantifying the spatial distribution of grassland AGB can provide useful information in herbivore carrying capacity and sustainable grassland management strategies.

Different methods were developed to estimate grassland AGB. One classical method is biomass harvesting, which is generally time‐consuming, destructive, and nonrepeatable over time (Briggs & Knapp, 1991; Singh, Lauenroth, & Steinhorst, 1975). Alternatively, satellite‐based estimation approach can nondestructively and effectively explore AGB patterns in both temporal scales and large spatial extents; this method combines remote sensing data with ground‐based observations (Jiang et al., 2015; Moreau, Bosseno, Gu, & Baret, 2003; Piao, Fang, Zhou, Tan, & Tao, 2007; Yang, Fang, Pan, & Ji, 2009). Even so, difficulty arises in accurate estimation of AGB by satellite‐based estimation approach (Gao, 2006). Another nondestructive approach is the using of plant cover assessment. Several studies reported a tight correlation between AGB and herb layer cover, and considered cover as a predictor of biomass. For example, Röttgermann, Steinlein, Beyschlag, and Dietz (2000) observed linear relationships between AGB and plant cover in low and open herbaceous vegetation. Flombaum and Sala (2009) directly employed plant cover to predict AGB for shrubs and grasses in arid ecosystems. Zhang, Cui, Shen, and Liu (2016) found that cover was tightly correlated with the aboveground, belowground, and total biomass at community level, and concluded that using cover to estimate shrub biomass can be applied in both arid ecosystems and alpine or subalpine environment. The linear relationships between plant cover and AGB for the less densely covered and rather simply structured vegetation types might lie in environmental stress, such as water, nutrient, or species growth form (Axmanová et al., 2012; Röttgermann et al., 2000). For dense and structurally diverse vegetation such as meadows, competition becomes more important, and communities are of various heights. Therefore, using cover as a surrogate for biomass might affect the results, and stand height should be considered during AGB estimation. Axmanová et al. (2012) proposed a model based on herb layer cover and plant median height to estimate AGB of different herbaceous types. Hence, it can be seen that the relationships between plant cover and AGB are not always linear, and the relationship can be influenced by factors of environment and communities.

Alpine grasslands are dominant and most widely distributed vegetation type on the Tibetan Plateau. Three main grassland types exist, namely desert steppe, alpine steppe, and alpine meadow, with coverage from open to dense. Alpine grassland AGB on the Tibetan Plateau was studied by quantifying spatial or temporal distribution from in situ measurements, model simulations, or remote sensing approaches (Luo et al., 2002; Shen et al., 2008; Tan et al., 2010; Yang et al., 2009; Yu, Wang, & Wang, 2010b; Yu, Zhou, Liu, & Zhou, 2010a). Vegetation cover in the large spatial extent of the plateau with various grassland types revealed distinct patterns and temporal changes by remote sensing detection (Meyer et al., 2017; Xu, Chen, & Levy, 2008). However, the relationships between cover and AGB for the alpine grasslands in northern Tibet are still not fully examined.

Understanding biotic and abiotic effects on AGB–cover relationship is important in accurately estimating AGB from cover. Several studies discussed possible environmental factors affecting AGB in grassland ecosystems. Biotic factors were associated with vegetation types, community structure, species richness, functional diversity, community composition, or dominant‐species traits within given experimental site (Cantarel, Bloor, & Soussana, 2013; Hector et al., 1999; Hooper & Vitousek, 1997; Ma et al., 2010; Rusch & Oesterheld, 1997; Tilman et al., 1997; Wang et al., 2012). Precipitation, temperature, and soil properties were considered major abiotic factors (Epstein, Lauenroth, & Burke, 1997; Sala, Parton, Joyce, & Lauenroth, 1988; Yang et al., 2009). A few studies have investigated correlations of AGB or vegetation cover with climate and soil properties in Tibetan Plateau (Jiang et al., 2015; Xu et al., 2008; Yang et al., 2009). However, these literatures lack information on how these factors influence AGB–cover relationships.

Influences of local or regional factors aforementioned are expected to depend on the study scale. However, the contributions of these factors to AGB–cover relationship at different scales were hardly seen in the previous studies. Such contributions were even less demonstrated for alpine grasslands in northern Tibet. Therefore, this study aims to answer the following questions: (i) how AGB of alpine grassland relates to plant cover at different spatial scales, and (ii) what the major factors influence AGB–cover relationship at different scales. Our first hypothesis states that AGB is not always linearly correlated to cover in northern Tibet because of the varying grassland densities. We explored the relationships using data sets of all grasslands and three grassland types as regional and landscape scales. Our second hypothesis proposes that aside from growth form and stands height, other factors, namely plant height, species composition, mean annual precipitation (MAP), and soil properties, could partially affect AGB–cover relationship of alpine grassland in northern Tibet. We expect that the effects of these factors vary across different grassland types. To verify these hypotheses, we investigated alpine grasslands by species and communities, and investigations differed at landscape to regional scales.

## 2 METHODS

### 2.1 Study area

**Figure 1:**
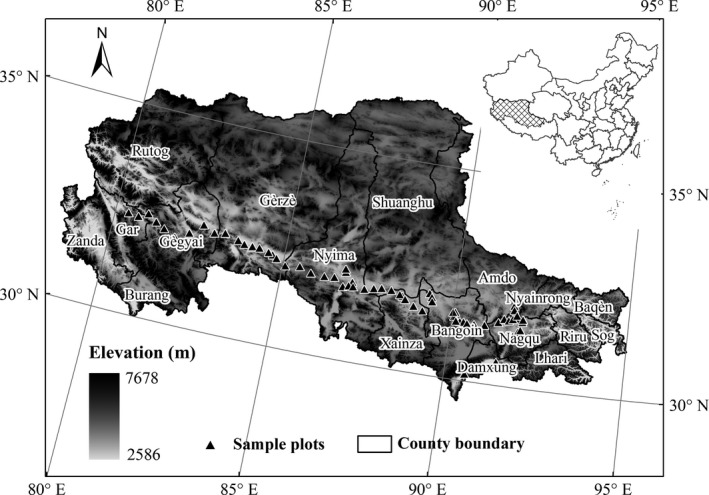
Study area and locations of 70 sample sites in northern Tibet

Study was conducted on northern Tibetan Plateau (locally named Changtang). Studied area is located at hinterland of the Qinghai–Tibet Plateau (29°53′–36°32′N; 78°41′–92°16′E) and covers 597,000 km^2^. Average elevation of study area exceeds 4,500 m (Figure 1). Annual precipitation ranges from 50 to 800 mm and decreases from southeast to northwest. Mean annual temperature is 6–10°C in the warmest month and below −10°C in the coldest month. Growing season usually begins in May and ends in September and lasts for 90–150 days. The most commonly distributed soil type is frozen calcium (alpine steppe soil) under alpine steppe, followed by alpine desert grassland soil and alpine desert soil under alpine semidesert and desert vegetation, respectively. Representative vegetation types are alpine meadows, which are composed primarily of *Kobresia pygmaea*; alpine steppes, which are principally composed of *Stipa purpurea*; desert grasslands, which are dominated by *Stipa glareosa* and *Ceratoides laten* (Li, 2000).

### 2.2 Data collection

We set up a west–east alpine grassland transect (the Northern Tibetan Plateau Alpine Grassland Transect) in May 2009 along a precipitation gradient. Transect cover ranges longitudes from 79.71 to 92.03°E and latitudes from 30.50 to 33.45°N and is approximately 1,200 km in length and 400 km wide (Li et al., 2011). Field surveys were conducted along the west–east transect at peak growing season (late July to August) from 2011 to 2012. A total of 70 sampled sites encompassed three main grassland types: alpine meadow, alpine steppe, and desert steppe. The geographical coordinates, elevation, and vegetation type were recorded at each site. Five 1 × 1 m plots were established randomly within each site. Community features and vascular species traits, including species name, cover, and natural height, were surveyed for each plot. Cover of individual species (species cover) and total cover of herb layer (community cover) within plots were estimated using 1 × 1 m quadrat divided into 100 0.1 × 0.1 m squares. All the sampled species were assigned to four plant functional groups (PFGs): cushions, graminoids, sedges, legumes, and forbs (other herbaceous species), and covers of each PFG were also calculated. To obtain AGB, we separately harvested aboveground standing biomass of each plant species in each plot in field. Then, this biomass was oven‐dried for 48 hr at 65°C and was weighed with a precision of 0.01 g in laboratory.

Five soil cores of 3.7 cm in diameter were collected from surface layer (0–5 cm) at each plot, mixed, and dried naturally and then brought to laboratory for total carbon and nitrogen content analyses. At the center of each 100 × 100 m site, three soil core samples with depth of 0 to 5 cm were obtained using cylindrical sampler (5.046 cm in diameter and 5 cm in length). Soil samples were weighed after oven‐drying at 105°C to constant weight and were further used to calculate bulk density (g/cm^3^). Soil bulk density was determined by dividing constant dried weight of soil samples by volume of cylindrical sampler (100 cm^3^). For carbon and nitrogen analyses, soil samples were sieved through 2 mm mesh, handpicked to remove plant detritus, and then ground in ball mill. Soil carbon content (%) and nitrogen content (%) were measured using elemental analyzer (vario MACRO cube, Germany).

MAP and mean annual temperature (MAT) were used as climatic variables. In obtaining MAP and MAT, we first acquired station records from 2011 to 2012 using database of China Meteorological Administration, which maintains 258 field observation stations in six provinces around the Tibetan Plateau. We selected monthly mean precipitation and temperature records from 200 stations with less than 5% of missing data during 2011–2012. The selected station climate records were then interpolated to spatial climate data sets with 1 × 1 km resolution using thin‐plate smoothing spline interpolation method of ANUsplin software package (Hutchinson, 2004), which has been proven to have a higher accuracy in mountain areas than other interpolation methods because it includes elevation as a covariate into the interpolation process (Hutchinson, 1995). Several studies involving our study regions obtained climatic data by applying this method and approved the accuracy (Chen et al., 2014; Tao et al., 2014). Thirdly, MAP and MAT in 2011 and 2012 were calculated respectively based on the monthly values. Lastly, MAP and MAT of the corresponding sampled year for each site were extracted in ArcGIS 10.0.

### 2.3 Statistical analyses

Two geographic scales were recognized: landscape and regional scales. Landscape scale encompassed three grassland types, which involved 22, 25, and 23 sampling sties, respectively. Regional scale represented entire gradient, including all 70 sampling sites.

Ordinary linear regression was employed to examine AGB–cover relationships of both community and species levels at regional scale of entire grassland and landscape scales of alpine meadow, alpine steppe, and desert steppe. Community covers corresponded to mean values for each sampling site. Meanwhile, species covers were mean values of individual species in all sampled plots. Hierarchical partitioning possibly alleviated multicollinearity problems, which were difficult or impossible to resolve using any common regression approaches (MacNally, 1996); it was employed to estimate independent contributions of each explanatory variable to AGB and community cover at both scales. Randomization tests (based on 100 randomizations) were used to estimate significance of independent contributions of each explanatory variable. Using partial correlation analysis with control for the effect of each biotic or abiotic factor, we could differentiate the importance of these factors on relationships between AGB and cover. The importance of a factor was determined by comparing the correlation coefficients difference with and without control for this factor.

All statistical analyses were implemented using R 2.14.0 (http://www.R-project.org). The R package “hier.part” and “rand.hp” were used for the hierarchical partitioning and randomization tests, respectively.

## 3 RESULTS

### 3.1 AGB and cover variations along the MAP gradient in transect

**Figure 2:**
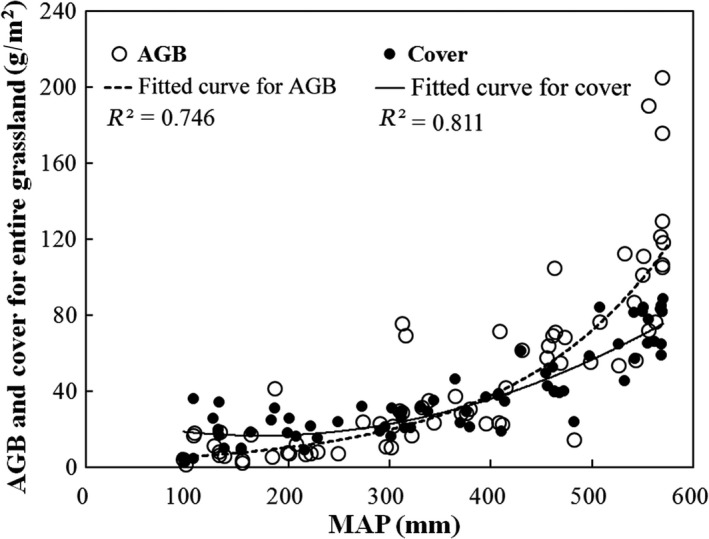
Grassland AGB and cover variations along a precipitation gradient in northern Tibet

Along the MAP gradient of 50–600 mm, AGB ranged from 1.1 to 204.7 g/m^2^, and cover ranged from 2.1% to 88.2% among 70 sampling sites (Figure 2). Mean AGB of entire grassland was 48.9 g/m^2^, with 100.4, 35.3 and 10.7 g/m^2^, and mean cover was 66.1%, 30.1% and 18.2% for alpine meadow, alpine steppe, and desert steppe, respectively (Table 1). A total of 112 plant species were then recorded within 70 sampling sites; plants belonged to 26 families, 56 genera. The dominant species were *K. pygmaea* and *S. purpurea*. Species richness in three grassland types decreased from 70 to 47 in alpine meadow to desert steppe.

**Table 1:** Sampled AGBs and species richness for various grassland types in northern Tibet

Grassland type	AGB (mean ± *SD*, g/m^2^)	Cover (mean ± *SD*, %)	Number of samples	Species richness
Alpine meadow	100.390 ± 42.867	66.134 ± 16.997	23	70
Alpine steppe	35.282 ± 20.095	30.136 ± 10.046	25	65
Desert steppe	10.661 ± 9.018	18.181 ± 9.830	22	47
Entire grassland	48.936 ± 46.565	38.207 ± 23.834	70	112

### 3.2 AGB–cover relationships for communities at regional and landscape scales

**Figure 3:**
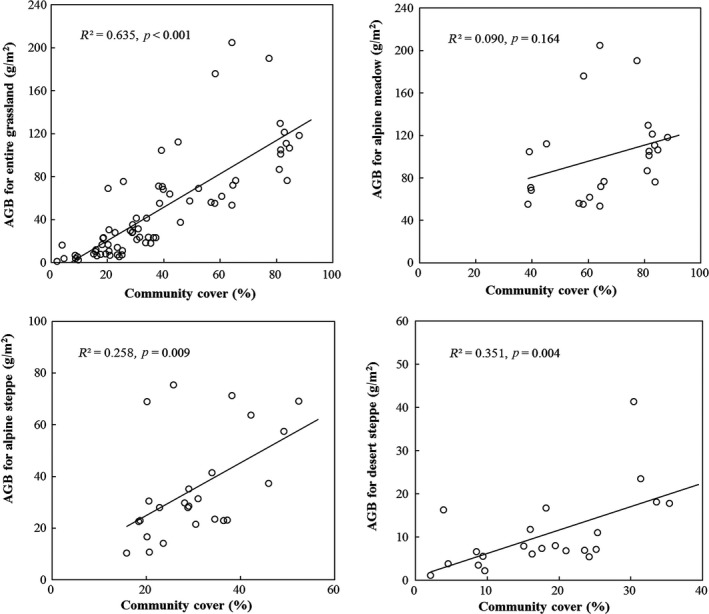
Relationships between AGB and community cover for entire grassland and three separate grassland types

Community cover holds positive linear relationships with AGB, both at landscape and regional scales (*p *<* *.01), but such relationship is not significant for alpine meadow (*p *>* *.05). Explanation ability of community cover varies across spatial scales with significantly lower *R* ^2^ for each grassland type than for entire grassland (Figure 3).

### 3.3 Species‐based AGB–cover relationships at different spatial scales

**Figure 4:**
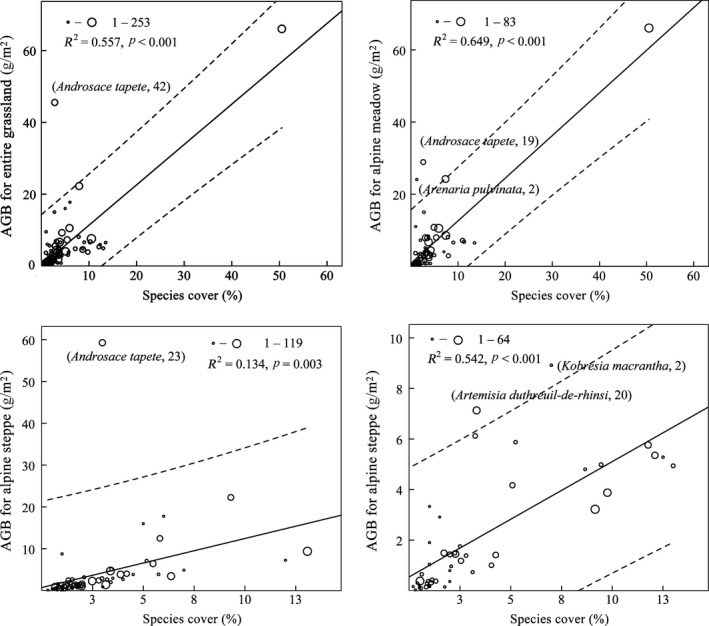
Relationships between AGB and species cover on basis of recorded species for entire grassland and three separate grassland types. Open circles represent frequencies of species present in grasslands. Size of open circle represents occurrence time of individual species in 350 plots. Species with confidence intervals outside 99% (dashed line) are shown with their occurrence times

Including all species present in 350 plots of 70 sampling sites, analysis centered on relationships between species cover and their AGB for entire grassland and specific grassland types (Figure 4). At both regional and landscape scales, AGB was linearly correlated to species cover (*p *<* *.01). Amount of variance explained by these linear relationships was 55.7% for AGB of all species present in entire grassland, 64.9% for alpine meadow, 13.4% for alpine steppe, and 44.4% for desert steppe. As shown in Figure 4, some species influenced AGB–cover relationship. *Androsace tapete* (Figure 4a,c) was far from fitted lines, *Arenaria pulvinate*,* Artemisia duthreuil‐de‐rhinsi*, and *Kobresia macrantha* were out of 99% confidence interval. *A. tapete* and *A. pulvinate* are cushion species.

### 3.4 Environmental factors influencing AGB, cover, and their relationships at two spatial scales

**Figure 5:**
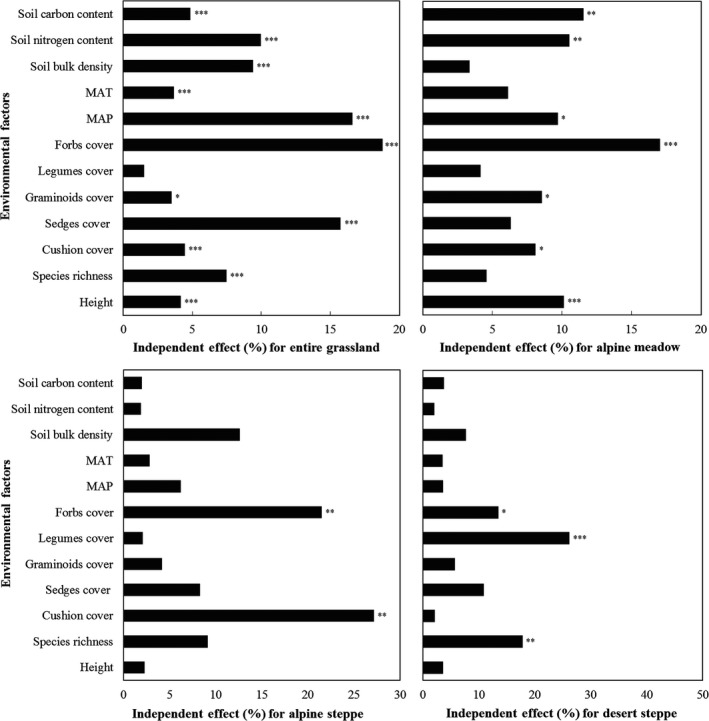
Hierarchical partitioning of environmental factors that illustrates the community AGB at different spatial scales in northern Tibet. Significances of independent contributions of each explanatory variable are indicated (***p <* *.001, ** .001 < *p *<* *.01, * .01 < *p *<* *.05)

**Figure 6:**
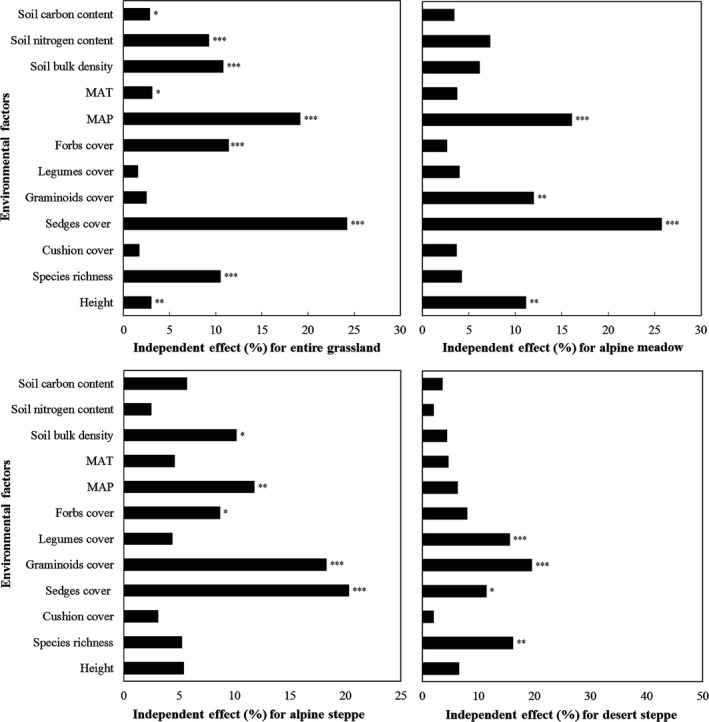
Contributions of environmental factors to the variations of community cover at different spatial scales in northern Tibet. Significances of independent contributions of each explanatory variable are indicated (***p <* *.001, ** .001 < *p *<* *.01, * .01 < *p *<* *.05)

The effects of biotic and abiotic factors including soil bulk density, soil carbon and nitrogen contents, MAP, MAT, cushions cover, sedges cover, legumes cover, graminoids cover, forbs cover, community height, and species richness on AGB and cover were quantified. Analyses revealed varied effects of these variables on AGB and cover at regional and landscape scales (Figures 5 and 6). We recognized that all factors significantly affected the regional AGB variation except for legumes cover, (*p *<* *.05), and MAP, forbs cover, and sedges cover contributed a great proportion of more than 50%. At landscape scale, determinants for AGB varied among different grassland types. Alpine meadow is significantly affected by stands height, forbs cover, and soil carbon and nitrogen content. Meantime, cushions cover and forbs cover significantly influenced alpine steppe, and species richness and legumes cover significantly affected desert steppe. For the variations of community cover, the contributions of these environmental factors were partly differed from those for AGB. For example, cushions cover did not significantly affect community cover for grasslands at any spatial scale, while graminoids cover only significantly influenced community cover for all three grassland types at landscape scale. However, landscape MAP was no longer an important factor for desert steppe, neither to AGB, nor to community cover.

Through partial correlation analyses, the importance of environmental factors to the relationship of AGB–cover were clarified and displayed in Table 2. At the regional scale, sedges cover and MAP were the two most important factors that shaped AGB–cover. The correlation coefficients were decreased to 0.383 and 0.393 with control for each of above factor, respectively, from initial coefficient of 0.797 without control for any factor. On the contrary, controlling for cushions cover increased the correlations coefficient. At the landscape scale, for alpine meadow, MAP, stands height, and sedge cover significantly affected the AGB–cover relationship, with the coefficients after controlling of −0.134, −0.077, and −0.057, respectively. For alpine steppe, the variations of coefficients were not so obvious, but it can be find out that cushion cover increased correlations coefficient once again. For desert steppe, legumes cover and species richness were the relative more important factors to the relationships of AGB–cover.

**Table 2:** Factors influenced AGB–cover relationships for three separate grassland types and entire grassland: two‐tailed partial correlation analysis with control of various environmental factors

Control factor	Entire grassland		Alpine meadow		Alpine steppe		Desert steppe	
	*r*	*p*	*r*	*p*	*r*	*p*	*r*	*p*
Height	.773	<.001	−.077	n.s	.506	.012	.565	.008
Speices richness	.701	<.001	.340	n.s	.437	.033	.378	n.s.
Cushion cover	.807	<.001	.237	n.s	.756	<.001	—	—
Forbs cover	.586	<.001	.319	n.s	.335	n.s.	.501	.021
Graminoids cover	.791	<.001	−.008	n.s	.515	.010	.568	.007
Legume cover	.798	<.001	.267	n.s	.523	.009	.352	n.s.
Sedges cover	.383	.001	−.057	n.s	.385	n.s.	.510	.018
MAP	.393	.001	−.134	n.s	.425	.038	.580	.006
MAT	.782	<.001	.254	n.s	.501	.013	.603	.004
Soil bulk density	.636	<.001	.193	n.s	.324	n.s.	.570	.007
Soil nitrogen content	.671	<.001	.073	n.s	.507	.011	.590	.005
soil carbon content	.786	<.001	.225	n.s	.515	.010	.571	.007
No control	.797	<.001	.300	n.s	.508	.010	.592	.004

n.s. not significant.

## 4 DISCUSSION AND CONCLUSION

### 4.1 Relationships between AGB and plant cover depend on spatial scale

Our results support our first hypothesis, suggesting that linkages between plant cover and AGB depended on spatial scales for grasslands in northern Tibet. Linear relationships existed in regional grassland at both community and species level. For communities at landscape scales, linear relationships were found within alpine and desert steppes, but without meadow. This aspect agrees with previous studies that significant linear relationships existed between AGB and plant cover, mostly for open and rather simply structured herbaceous vegetation (Axmanová et al., 2012; Muukkonen et al., 2006; Röttgermann et al., 2000). In present study, spatial scale directly determines spatial extent and somehow affects ranges of plant cover. To a large extent, species composition and plant cover were widely variable. Three individual grassland types had lower *R* ^2^ values than that for entire grassland, which were probably due to limited ranges of community cover encountered in these subsets. It is verified our first hypothesis that AGB is not always linearly correlated with cover in northern Tibet because of the varying grassland densities.

### 4.2 Multiple influences on AGB–cover relationship

Species composition potentially affected AGB–cover relationship. At species level, species, such as cushion plant *A. tapete*, highly influenced explanation of cover to AGB variance. Cushion plants are compact, low‐growing, and mat‐forming. These plants are commonly found in alpine, subalpine, arctic, or subarctic environments globally. Cushions plants also possess relatively large and deep tap roots with life histories adapted to slow growth in nutrient‐poor environment with delayed reproductivity and reproductive‐cycle adaptations (Malcolm & Malcolm, 1988). Thus, cushion plants possess relatively higher AGB than those of other plant types. Cushions cover significantly affected total AGB but only contributed little to total community cover of all cushion‐grown grassland types. Consequently, the cushions cover affected the relationship of AGB–cover for alpine steppe, where cushions mostly occurred. The influences of other species or PFGs were also related to their growth traits and distribution in the grassland. For instance, sedges and graminoids distribute in all grassland types, but their dominance is different, with sedges being dominant in meadows, and graminoids being dominant in steppes. Sedges, especially the most dominant species, *K. pygmaea* is relatively much shorter height than graminoids in height, where gramimoids have dispersed life‐form and hollow stems. This effect indicated that species composition or PFG type was an important factor affecting AGB–cover relationship through affecting their AGB or covers, and such result was in accordance with those of Chaneton, Lemcoff, and Lavado (1996), who claimed that changes in life‐form composition primarily influenced processes related to production.

Different species composition may form high species richness. Classical theory predicted that species‐rich communities were more productive than less diverse communities. This observation may be explained by higher number of species in former; this numbers varies in spatial and temporal resource capture and maximizes complete use of resource (Rusch & Oesterheld, 1997). In this study, species richness influence on AGB or cover which depended on grassland types. This aspect significantly affected AGB and cover for desert steppe, and then affected the relationship between AGB and cover. Higher species richness indicates a more complete use of available water and nutrients because of niche complementarity (Bai, Han, Wu, Chen, & Li, 2004; Caldeira, Hector, Loreau, & Pereira, 2005; Rusch & Oesterheld, 1997). For less competition communities, such as desert steppe, more species means higher cover and more productivity.

Stands height proved to be a complementary parameter for estimating AGB from cover (Axmanová et al., 2012), because cover of herb layer has an upper limit of 100%, which may lead to misinterpretation of AGB for dense and structurally diverse vegetation. Consistently, plant height obviously affects correlation between AGB and cover only for alpine meadow, the most dense and complicatedly structured grassland type in the study region.

MAP, soil bulk density, and soil carbon and nitrogen content were important environmental factors that reflect water and nutrient availability. They can explain a great proportion of regional AGB and cover variation at the regional scale, but were not that important to individual grassland type at landscape scale. Many studies illustrated that AGB of grasslands is sensitive to precipitation at regional or broad scales (Bai et al., 2004, 2008; Hsu, Powell, & Adler, 2012; Jiang et al., 2017; Knapp & Smith, 2001; Ni, 2004), and normally positive correlation between MAP and AGB was presented (Sala, Gherardi, Reichmann, Jobbagy, & Peters, 2012). AGB and cover both increased along MAP in this study. MAP is primary factor limiting vegetation growth, and determines plant cover and species composition (Guo et al., 2012; Paruelo, Lauenroth, Burke, & Sala, 1999). This factor significantly influenced the regional AGB and cover pattern in northern Tibet. Spatial sensitivity of AGB to MAP was distinct among grassland types, with only alpine meadow that showing sensitivity. MAP also affected community cover variation at the alpine steppe, instead of at the desert steppe. The distinct effects were probably due to different water‐use strategies among grasslands with varied species compositions, compensatory effects in communities with high species richness (Bai et al., 2004; Guo et al., 2012), and various soil types ecompassed.

Soil properties, including bulk density, and carbon and nitrogen contents are directly influenced by soil type. Soil type in northern Tibet changes along west–east transect from alpine desert grassland soil and frozen calcium (alpine steppe soil) to cold frozen felt soil (alpine meadow soil) (Li, 2000). Soil texture of desert steppe is coarse, whereas texture of meadow loamy soil is fine and with higher organic matter content. Soil texture and bulk density directly affected water‐holding capacity and water availability for plant growth. Total nitrogen and carbon contents are closely related to nitrogen availability in grassland ecosystems; thus, these elements can serve as proxies for nitrogen availability (Bai et al., 2008; Vitousek & Howarth, 1991). High nutrient levels, including high nitrogen and carbon contents, favor fast‐growing competitive species and cause damage in low‐ and slow‐growing ones (Merunková & Chytrý, 2012). In nutrient‐poor environment, linear relationships between AGB and plant cover were found by Röttgermann et al. (2000). It is striking that the consistent result was obtained in our study for desert steppe and alpine steppe with limited resources. These resources did not directly affect the relationships between AGB and cover, but determined the species and communities distribution, together with climatic variables. In view of this, the result only supports part of our second hypothesis: aside from growth form and stands height, covers of PFGs and MAP partially affect AGB–cover relationship of alpine grassland in northern Tibet.

## 5 CONCLUSIONS

We conclude that the relationships between AGB and cover are generally positive and linear at both regional and landscape scales in northern Tibetan grasslands. Spatial scale may affect ranges of cover and then change influence of cover on AGB. AGB–cover relationships were influenced by many other variables. Cover of cushion plants and sedges, and MAP were importantly controlled AGB–cover relationship at regional scale. At landscape scales, factors that affected the relationship were differed for grassland types. MAP, cover of sedges and graminoids, and stands height were important factors for alpine meadow; cushions cover increased the correlation coefficient of AGB and cover in alpine steppe; species richness and legumes cover affected desert steppe much. Herein, the relationships between AGB and cover at different spatial scales likely most depend on community composition of different functional groups. In future deriving AGB patterns at different spatial scales, it is necessary to consider community composition to obtain acceptable accuracy.

## CONFLICT OF INTEREST

None declared.

## AUTHOR CONTRIBUTIONS

Jiang Y. designed study, conducted this research, collected field data, performed data analysis, and drafted manuscript. Zhang Y. conceived, designed, and coordinated study, and revised the manuscript. Wu Y. analyzed and interpreted data. Hu R. revised the manuscript, whereas Zhu J., Tao J., and Zhang T. collected field data. All authors contributed to final approval for publication.
